# Evaluation of the Liat Cdiff Assay for Direct Detection of Clostridioides difficile Toxin Genes within 20 Minutes

**DOI:** 10.1128/JCM.00416-19

**Published:** 2019-05-24

**Authors:** David J. Hetem, Ingrid Bos-Sanders, Roel H. T. Nijhuis, Sven Tamminga, Livia Berlinger, Ed J. Kuijper, Joanna J. Sickler, Eric C. J. Claas

**Affiliations:** a Haaglanden Medical Center, Department of Microbiology, The Hague, The Netherlands; b Leiden University Medical Center, Department of Medical Microbiology, Leiden, The Netherlands; c BioAnalytica AG, Department of Microbiology, Luzern, Switzerland; d Roche Molecular Systems, Pleasanton, California, USA; Brigham and Women's Hospital

**Keywords:** *Clostridioides difficile*, Liat, PCR, GeneXpert

## Abstract

Clostridioides difficile is the main causative agent of antibiotic-associated diarrhea. Prompt diagnosis is required for initiation of timely infection control measures and appropriate adjustment of antibiotic treatment. The cobas Cdiff assay for use on the cobas Liat system enables a diagnostic result in 20 minutes. A total of 252 prospective (*n* = 150) and retrospective (*n* = 102) stool specimens from The Netherlands, France, and Switzerland were tested on the cobas Cdiff assay using the Xpert C. difficile assay as a reference method. The overall positive and negative percent agreement (PPA and NPA, respectively) of the cobas Cdiff assay compared with the Xpert C. difficile assay was 98.0% (100/102; 95% confidence interval [CI], 93.1% to 99.5%) and 94.0% (141/150; 95% CI, 89.0% to 96.8%), respectively. When comparing the PPAs of cobas Cdiff and Xpert C. difficile with culture, the results were 91.7% (55/60; 95% CI, 81.9% to 96.4%) and 85.0% (51/60; 95% CI, 73.9% to 91.9%), respectively. The difference was not statistically significant. The cobas Cdiff assay offers a very rapid alternative for diagnosing C. difficile infection. The 20-minute turnaround time provides the potential for point-of-care testing so that adequate infection control measures can be initiated promptly.

## Abstract (precis)

Clostridioides difficile is the main causative agent of antibiotic-associated diarrhea. Prompt diagnosis is required for initiation of timely infection control measures and appropriate adjustment of antibiotic treatment.

## INTRODUCTION

Clostridioides difficile is an anaerobic Gram-positive spore-forming bacillus and a leading cause in nosocomial infectious diarrhea (1, 2). Symptoms of C. difficile infections (CDIs) can range from mild self-limiting diarrhea to life-threatening pseudomembranous colitis, toxic megacolon, and death. CDI is associated with an increased length of hospital stay and increased health care expenses (3). Colonization with C. difficile, without clinical symptoms, is suggested as a potential source of transmission, although screening for asymptomatic carriage and contact precautions are not yet recommended (4). C. difficile carriage in healthy adults without clinical symptoms ranges from 4% to 15%, being higher in neonates (4% to 71%) and hospitalized patients (3% to 21%) (5).

Rapid diagnosis of CDI is important to initiate the proper infection control measures and timely start of an appropriate antibiotic treatment. Current guidelines recommend multistep algorithms for the diagnosis of CDI (4, 6), with primary screening for CDI using a test with a high negative predictive value, such as nucleic acid amplification tests (NAAT) or glutamate dehydrogenase (GDH) enzyme immune assays (EIAs). If positive, the actual presence of free fecal toxins should be determined using a test with a high positive predictive value (e.g., toxin A/B EIA). The presence or absence of free fecal toxins is considered an important tool to differentiate colonization from infection. NAAT-detecting toxin-producing genes are becoming more popular as a first step screening, given their ease of use and high sensitivity (7, 8).

In some countries, testing for C. difficile is performed in centralized laboratories using batch processing of specimens, lengthening the time to result. Decentralized testing and point-of-care solutions can potentially reduce the time to result for C. difficile testing. Previous studies have shown the benefit of faster diagnostic results for patient management (9, 10). The cobas Cdiff test for use on the cobas Liat system manufactured by Roche Diagnostics (Pleasanton, CA, USA) enables point-of-care testing and a diagnostic result in 20 minutes. The Liat is a compact PCR system designed for on-demand testing in point-of-care settings, such as physician clinics, pharmacies, hospital laboratories, and satellite laboratories (11).

In this study, we evaluate the performance characteristics of the Liat system by comparing it with the Cepheid Xpert C. difficile (Xpert) toxin genes diagnostic assay manufactured by Cepheid (Sunnyvale, CA, USA).

## MATERIALS AND METHODS

A prospective study was conducted using predominantly unformed stool specimens that were submitted to the clinical microbiology laboratory of the Leiden University Medical Center (LUMC) for diagnosis of C. difficile infection. Following the routine diagnostic workflow, a laboratory-developed, internally controlled, real-time PCR assay (LDT) (7) was used to screen for the C. difficile toxin B gene. From January to September 2017, 75 C. difficile-positive specimens were included in the study. For each positive sample, a negative fecal specimen was included. The study received a waiver from the institutional review board because all samples were deidentified without using patient characteristics based on an established procedure at LUMC.

PCR-positive samples were also cultured, and all PCR-positive specimens were subjected to the Vidas C. difficile toxin A&B test (bioMérieux, Marcy l'Etoile, France). The presence of free feces toxin by Vidas antigen detection was considered indicative for CDI. Of each included fecal sample, three aliquots were stored at −20°C until further testing using the Liat and Xpert assays. Culture-positive specimens were also subjected to PCR ribotyping, as described previously (12).

In addition, a selection of 25 positive and 25 negative neat fecal specimens were obtained from Laboratiore Cerba in Saint-Ouen-l'Aumône, France (obtained by Roche) and from BioAnalytica in Luzern, Switzerland. The latter collection consisted of fecal specimens in Cary-Blair medium, enabling analysis of the performance of the rapid testing devices on this matrix as well. No ribotyping or culture was performed on these samples collected retrospectively.

### Enriched culture.

A 0.5-g portion of feces was suspended in 0.5 ml 96% ethanol, incubated for an hour, and, subsequently, 1 drop was cultured anaerobically on plates with selective medium containing cefoxitin, amphotericin B, and cycloserine (CLO) and Columbia agar with colistin and nalidixic acid (CNA). For enriched culture, a preincubation cycloserine-cefoxitin-mannitol broth with taurocholate, lysozyme, and cysteine (CCMB-TAL) was performed.

### *tcdB* PCR (LDT).

Half a pea or 200 µl of fecal material was resuspended in 1 ml of stool transport and recovery (STAR) buffer (Roche Diagnostics, Pleasanton, CA, USA) with Precellys beads (Bertin, Montigny-le-Bretonneux, France). After mixing for 5 min at 2,200 rpm, the sample was left for 5 minutes and subsequently centrifuged for 1 minute at 14,000 rpm. Then, 200 µl of the supernatant was subjected to nucleic acid extraction using the Viral NA small volume kit on a MagNApure 96 system (Roche Diagnostics) in the presence of a phocine herpesvirus (PhHV) internal control (IC), yielding a final eluate of 100 µl. Finally, the *tcdB* gene was amplified from 10 µl of eluate, as described previously (7).

### Cobas Cdiff test.

The procedure was performed according to the manufacturer’s instructions. A tip of a polyester swab was immersed in the stool specimen and then transferred to the cobas PCR medium tube. After breaking the swab, the medium was vortexed for 5 seconds, after which 200 µl of the sample was pipetted into the Liat assay tube. Subsequently, the test was run as instructed.

### *Cepheid Xpert* C. difficile.

The procedure was carried out according to the manufacturer’s instructions. The swab was immersed in feces and inserted into the sample reagent tube provided. After breaking the swab and closing the lid, the sample was vortexed for 10 seconds. Next, the complete sample was pipetted into the sample chamber of the Xpert cartridge. After transferring the cartridge to the GeneXpert instrument, the run was started. All positive samples with quantitation cycle (*C_q_*) of >37 were called negative by the GeneXpert software.

## RESULTS

Altogether, the total sample size was 252 samples, namely, 150 for the prospective part (75 positive, 75 negative) and 102 (50 positive, 52 negative) for the retrospective part of the study.

### Prospective study.

**FIG 1:**
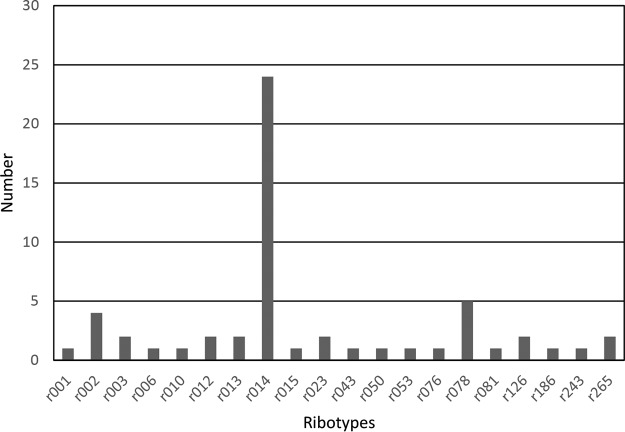
PCR ribotypes obtained from the prospective study.

Based on the *tcdB* screening by LDT, 75 positive samples were selected to be included in the study, with *C_q_* values ranging from 19.2 to 39.5. Of these 75 positive samples, 19 were positive for the presence of free toxins and diagnosed as CDI and 14 were indeterminate. Culture was performed on all positive specimens irrespective of *C_q_* value, resulting in 60 culture-positive C. difficile isolates. PCR ribotype 014 was the most prevalent (*n* = 24), followed by PCR ribotype 078 (*n* = 5) and PCR ribotype 002 (*n* = 4). Altogether, 20 different PCR ribotypes, but no PCR ribotype 027, were detected in this study group (Fig. 1).

Subjecting all 150 samples to both the Liat and the Xpert procedures showed good overall concordance between the assays at 95.3% (143/150; 95% CI, 90.7% to 97.7%) (Tables 1 and 2).

**TABLE 1:** Positive and negative test results for clinical samples by cobas Liat Cdiff versus Xpert PCR and culture

Test result by specimen type	Liat	
	+	−
Xpert		
Prospective		
+	53	1
−	6	90
Retrospective		
+	47	1
−	3	51
All samples		
+	100	2
−	9	141
Culture		
Prospective		
+	55	5
−	4	73

**TABLE 2:** Comparison of the cobas Liat Cdiff and Xpert PCR tests^*a*^

Comparison measure	Values by specimen type					
	Prospective		Retrospective		All	
	Estimate (% [*n*])	95% CI (%)	Estimate (% [*n*])	95% CI (%)	Estimate (% [*n*])	95% CI (%)
PPA	98.1 (53/54)	90.2–99.7	97.9 (47/48)	89.1–99.6	98.0 (100/102)	93.1–99.5
NPA	93.8 (90/96)	87.0–97.1	94.4 (51/54)	84.9–98.1	94.0 (141/150)	89.0–96.8
PPV	89.8 (53/59)	79.5–95.3	94.0 (47/50)	83.8–97.9	91.7 (100/109)	85.1–95.6
NPV	98.9 (90/91)	94.0–99.8	98.1 (51/52)	89.9–99.7	98.6 (141/143)	95.0–99.6
OPA	95.3 (143/150)	90.7–97.7	96.1 (98/102)	90.4–98.5	95.6 (241/252)	92.4–97.6

a CI, confidence interval; NPA, negative percent agreement; NPV, negative predictive value; OPA, overall percent agreement; PPA, positive percent agreement; PPV, positive predictive value.

Culture was performed on 137 of the prospective samples to support discrepancy analysis between the two assays. Compared to culture, the PPAs of Liat and Xpert were 91.7% (55/60; 95% CI, 81.9% to 96.4%) and 85.0% (51/60; 95% CI, 73.9% to 91.9%), respectively, with negative predictive values of 93.6% (73/78; 95% CI, 85.9% to 97.2%) and 89.2% (74/83; 95% CI, 80.7% to 94.2%). The difference was not statistically significant, with a *P* value of 0.39 from 2-sample chi-square test for testing equal PPAs with continuity correction. The NPAs were 94.8% (73/77; 95% CI, 87.4% to 98.0%) and 96.1% (74/77; 95% CI, 89.2% to 98.7%) for Liat and Xpert, respectively, when compared with those of culture.

For the seven prospective discordant samples, five culture results matched the Liat test (Table 3). Four of the six Liat positive/Xpert negative samples recorded Xpert threshold cycle (Ct) values above the 37 cutoff, ranging from 37.2 to 38.8. Vidas results were available for six of the seven discordant samples. Six were negative and one was indeterminate.

**TABLE 3:** Cobas Liat Cdiff and Xpert discordant prospective samples

Category	Population	Sample number	Liat		Xpert		Direct culture result
			Result	*C_T_*^*a*^	Result	*C_T_*	
False positive	Prospective	418-135	+	30.42	−		−
True positives		418-156	+	33.07	−	37.9	+
		418-173	+	33.21	−	37.2	+
		418-185	+	20.38	−		+
		418-201	+	30.55	−	38.8	+
		418-237	+	30.37	−	38	+
False negative	Prospective	418-167	−		+	37.6	+

a *C_T_*, cycle threshold.

### Retrospective study.

The 102 retrospective samples also demonstrated good overall concordance between the Liat and the Xpert assays at 96.1% (98/102; 95% CI, 90.4% to 98.5%) (Tables 1 and 2).

The overall PPA and NPA between the two assays, including both prospective and retrospective samples, were 98.0% (100/102; 95% CI, 93.1% to 99.5%) and 94.0% (141/150; 95% CI, 89.0% to 96.8%), with a negative predictive value of 98.6% (141/143; 95% CI, 95.0% to 99.6%) (Table 1).

## DISCUSSION

This is the third study comparing the performance of the Liat test to routine laboratory NAAT for the detection of C. difficile. It is the first study in Europe and the first to compare Liat with the *Xpert* C. difficile assay. The inclusion of samples from the French and Swiss populations increases the geographic diversity of the sample set to make the findings relevant beyond The Netherlands. The test had strong agreement with Xpert for prospective and retrospective samples, with an overall PPA of 98.0% and NPA of 94.0% for all samples. These findings are in line with the results from other published studies. A prospective comparison between the Liat and Xpert C. difficile/Epi test on fresh samples in the US population found 94.5% and 98.0% positive and negative percent agreement (13). A second prospective study in the U.S. population using the cobas 4800 Cdiff as the reference yielded 95.4% and 99.4% PPA and NPA, respectively (11).

Culture results for the discrepant analysis found that most agreed with the Liat result (five of seven) for prospective samples. However, the clinical relevance of samples near the limit of detection and/or cutoff for PCR assays remains unclear. None of these samples were toxin A/B positive in the Vidas test, possibly indicating colonization with C. difficile rather than infection.

PCR ribotyping enables differentiation of C. difficile strains by amplification of the 16S-23S intergenic spacer regions. This is not only useful for epidemiology but also for identification of hypervirulent 027 and 078 ribotypes. Obviously, all ribotypes need to be detected by diagnostic methods and all ribotypes encountered in this study, including some new ribotypes, could be detected by Liat.

The LDT used routinely at LUMC was the screen to select positive and negative samples for the study. This test had a significantly higher positivity rate than either Liat (59/75) or Xpert (54/75). The LDT used for screening had no cutoff and as a result continued to amplify the bacterial DNA to make the detection of very low levels of C. difficile possible, likely explaining the high number of positives compared with Liat and Xpert as well as culture. Both Liat and Xpert have a cutoff built into the algorithm. Setting this cutoff is done to balance sensitivity, specificity, and turnaround time (TAT) of the assay. The additional LDT-positive samples had *C_q_* values ranging from 32 to 39, and all were negative in the toxin assay.

The study has several limitations. First, the samples were aliquoted and different aliquots were used for each test. Variability due to uneven distribution of the pathogen within the samples may have impacted results. In addition, all samples went through a freeze/thaw process before PCR testing and so were not fresh, as indicated by the instructions for use for both assays. The samples in the prospective portion were not sequential and were included in the study based on a screening that created a 50% prevalence rate not reflective of the general population. Lastly, samples were deidentified and so clinical information on the patients was not available for the analysis. This made it impossible to look at the diagnostic implications for PCR-positive and toxin A/B-negative samples.

A strength of the study was utilizing samples from multiple geographic areas in Europe to demonstrate a high level of concordance. Additionally, different fecal matrices were used in the study, indicating the wide application of the assay. In addition, the availability of a toxin A/B and culture result for the prospective studies enables assessment of how the assay could impact clinical decision making. It also highlights the ongoing diagnostic challenges of distinguishing between acute CDI and C. difficile colonization.

The key finding is that the fast TAT of 20 minutes demonstrated by the Liat assay does not decrease the sensitivity of the test. The typical paradigm is to assume that increased speed and ease of use can require a performance compromise for the benefit of these features. The results of this study indicate the Liat assay still delivers the performance of routine laboratory NAAT with the potential to increase the speed and access to test results. This feature makes it a valuable assay for health systems to consider utilizing for C. difficile testing.
